# Measuring Goal Progress Using the Goal‐Based Outcome Measure in Youth Wellness Hubs Ontario—An Integrated Youth Services Network

**DOI:** 10.1111/hex.70240

**Published:** 2025-03-26

**Authors:** Deb Chiodo, Aaron Turpin, Janis Wolfe, Karleigh Darnay, Jo Henderson

**Affiliations:** ^1^ Youth Wellness Hubs Ontario (YWHO) Toronto Ontario Canada; ^2^ Department of Human Services and Early Learning MacEwan Univesity Edmonton Alberta Canada; ^3^ Department of Psychiatry University of Toronto Toronto Ontario Canada

**Keywords:** goals, Integrated Youth Services, mental health, youth

## Abstract

**Background:**

Goal‐based outcomes (GBOs) are a tool for youth‐directed care and have been successfully used in a small number of community youth mental health settings. Youth Wellness Hubs Ontario (YWHO) uses GBO data provided by youth to deliver care that amplifies youth voice in decision‐making about supports that are important and meaningful to them.

**Objective:**

This article summarizes GBO data from youth receiving clinical and nonclinical services at YWHO hubs to develop a more nuanced understanding of the type of goals set by young people when accessing services.

**Design:**

A quantitative design utilizing content analysis was adopted to organize goal data into descriptive categories, which were further analysed by goal distribution and mean rating scores.

**Participants:**

Youth (*n* = 1851) from 22 YWHO networks provided GBO and demographic data. Data were collected across 7348 service visits and youth provided 19,290 goals. Consent for service was obtained, which included the use of personal health information to monitor progress, quality improvement and for evaluation purposes.

**Variables:**

GBO data included a written goal and scaled goal rating component. Service visits and demographic variables were tabulated, whereas a mean score for goal achievement (i.e., goal rating) was generated.

**Results:**

Six themes emerged from the analysis, including *improve mental health* (42.7% coverage, mean rating 3.9), *connect to services* (20.9% coverage, mean rating 4.4), *intrapersonal development* (15.3% coverage, mean rating 3.1), *interpersonal development* (12.0% coverage, mean rating 3.5), *improve physical health* (5.4% coverage, mean rating 2.9) and *address substance use/other addiction* (3.0% coverage, mean rating 3.1).

**Conclusion:**

The GBO tool allows youth to actively participate in setting their own assessment and outcome criteria, indicate which areas they require support and wish to improve and personalize shared decision‐making.

**Patient or Public Contribution:**

The YWHO model was co‐developed by youth, families, service providers and researchers. Youth and families informed the data measures and collection processes described within this article.

## 1 Introduction

The prevalence of major mental health and substance use challenges is disproportionately high in youth between the ages of 12 and 24 in Canada [1, 2], and is linked to several functional impairments, including adverse social, emotional, physiological and psychological outcomes [3]. Transition‐aged (18–25 years old) youth often face increased adverse health and mental health challenges due to a lack of developmentally appropriate services [4, 5, 6]. Access to timely, effective and youth‐centred treatment is greatly needed [7] but often hindered by a fragmented service system that is poorly connected and lacks developmentally appropriate and rigorously evaluated services [8, 9]. Globally, Integrated Youth Services (IYS) is a movement to transform care for young people through a community‐based approach, whereby service providers from various disciplines and sectors come together, often in the same space, to provide holistic care for youth and their families [9, 10, 11, 12, 13, 14]. IYS focuses on early intervention, enhancing access and delivering youth‐friendly services that are driven by youth needs [7, 13, 15], with the goal of reducing system fragmentation by creating a network of service providers with positive relationships and collaborative values in supporting positive youth outcomes [7, 10, 11].

Centre to the values of IYS is the active participation of youth, family and community members in decision‐making processes throughout the implementation of the model [11, 16, 17]. This may be facilitated in various ways, including engaging youth as active decision‐makers in their own care, the development of advisory committees or circles and the regular collection of data aimed at monitoring the progress of outcomes that are clinically and personally relevant—that is, recovery‐oriented, sensitive to developmental phases and based on the personal goals of youth [17, 18]. Early research on IYS systems in North America has linked implementation to increased positive youth outcomes including service access, overall functionality and service satisfaction [11, 19], and increased staff mental health literacy and commitment to serving youth [20]. The current paper builds on the IYS knowledge base by reporting on GBO data from across a network of IYS hubs in Ontario, Canada. It asks the following question: (1) *What are the types and frequencies of goals set by youth while engaged at Youth Wellness Hubs Ontario?*

## 2 Goal‐Based Outcomes (GBOs)

GBOs provide a measure of youth functionality by allowing youth to articulate their own service‐related needs [21], often through inquiring about pertinent goals directly with youth at the outset of the meeting. GBOs are known as idiographic patient‐reported outcome measures (PROMs), and are found to support the process of creating data that are closer to a youth's own interpretation of their needs [22, 23]. In contrast, standardized instruments, which are often used to measure the effectiveness of community mental health and substance use treatment, tend to be inflexible to the co‐occurring and contextual biopsychosocial challenges and priorities faced by youth [24]. The use of GBOs is found to support what is termed ‘patient involvement and engagement’ by capturing the dynamic range of needs of youth in situ during service provision [21] and has been positively associated with youth service engagement [25]. Similar tools, such as the Bern Inventory of Treatment Goals (BIT‐G [26]), offer a goal taxonomy spanning psychological and existential domains, encompassing concerns such as coping with specific problems, interpersonal dynamics, well‐being, existential contemplation and personal growth.

Early formative GBO research has worked towards developing a typology of goal categorization when using the GBO tool. For example, O'Reilly, McKenna, and Fitzgerald [24] applied a mixed‐methods analytical approach to GBO data provided by youth engaged in service hubs in Ireland. Results organized goal data into three major themes (i.e., developing coping mechanisms, personal growth and managing interpersonal difficulties) and revealed clinically significant increases in goal achievement across these domains [24]. Similarly, Rupani et al. [27] highlighted discrepancies between young people's goals and counsellor‐reported issues in a school‐based counselling programme, emphasizing the importance of aligning counselling with youth's specific therapeutic needs for improved outcomes. In this study, youth primarily focused on goals related to improving self‐confidence and reducing anxiety and counsellors often emphasized contextual issues, particularly family relationships [27].

The GBO measure has been used in a small number of IYS settings in Canada and has been linked to increased support for mental health and well‐being outcomes in youth [28, 29]; however, no study to date within the IYS context has investigated youth's self‐identified goals for intervention, despite calls to increase relevance and engagement of interventions for young people based on goals that youth themselves identify [29].

## 3 Youth Wellness Hubs Ontario (YWHO)

Data reported in this article have been collected from youth receiving services across Ontario through hubs operated as part of YWHO (see [7] for a full description of the YWHO model). YWHO is an IYS network consisting of hubs in over 35 geographically diverse Ontario communities, operated by 27 government‐funded networks. Youth ages 12–25 years can access hubs to address a continuum of needs ranging from mental health, substance use health, primary care, social and community‐based supports, and wellness and health promotion programming and services. Service interventions, determined in part through the use of standardized clinical screeners and service goals identified by youth, include low‐intensity options such as psychoeducation and solution‐focused brief therapy, moderate intensity services such as structured psychotherapy for mental health concerns (motivational interviewing [MI], cognitive behavioural therapy [CBT], 6–12 sessions), and high‐intensity services such as psychiatric or addiction medicine services [7]. Services are provided virtually and in‐person by teams of interdisciplinary hub staff and focus on evidence‐based and evidence‐generating, low‐barrier approaches to addressing health and social needs experienced by youth. Service offerings differ across hubs according to localized needs and resources, are often guided by broader community planning efforts and typically include youth and family advisory committees as well as local hub network governance tables. YWHO operates as a Learning Health System (LHS; [30]) where networks collectively drive system change through continuous learning and growth by bringing together information from practice, evaluation, quality improvement and research and feeding it back to the system in ways that are meaningful and useful. YWHO networks are supported with implementation, evaluation, clinical, operational, equity and strategic support from the YWHO Provincial Office (YWHO‐PO) hosted by the Centre for Addiction and Mental Health (CAMH) in Toronto, Canada.

Youth self‐reported goals are utilized by YWHO hub staff to facilitate service provision and ensure interventions are responsive to a youth's current needs. The GBO measure is part of a broader measurement‐based care model at YWHO that helps track individual youth's progress toward self‐identified goals and related outcomes and prompts staff to explore new and ongoing challenges and objectives that youth are working to address. The measurement‐based care process also includes the collection of youth sociodemographic data, which, together with multidimensional goals and wellness measures, supports personalized approaches to youth needs. As a provincial network committed to improving youth outcomes, the YWHO‐PO also gathers outcome data (which includes GBOs) at the hub and provincial levels to support quality improvement and identify areas of strength and gaps for future development within communities. This article reports on data regarding the types of goals set by youth as well as self‐reported goal achievement scores.

## 4 Materials and Methods

### 4.1 Procedure

A quantitative approach utilizing content [31] and descriptive analyses was adopted to answer the research question. GBO data, along with demographic information, were gathered routinely as part of service provision at YWHO hubs. Analyses were conducted as part of YWHO's commitment to continuous quality improvement and were reviewed and approved by CAMH's Quality Project Ethics Review Committee (#QPER_47).

### 4.2 Participants

Participants included youth (*n* = 1851) aged 12–25 years receiving services at YWHO between April 2020 and March 2023. At the beginning of a visit, youth complete a demographic form, the GBO measure (‘GBO’), and standardized clinical screeners if youth are requesting mental health or substance use support. The GBO tool is completed by youth for either clinical or nonclinical services, and informed consent for quality improvement data use is obtained. Responses are collected via a youth‐friendly electronic device (i.e., iPad) while youth are waiting to see a service provider, or 24 h in advance of an appointment if youth are seen virtually. A Youth Wellness Facilitator, Peer Support Worker and/or other hub staff are available during the consent process and data collection to support youth and answer any questions.

YWHO Networks operate a robust data platform that facilitates measurement‐based care, personalized treatment plans and system‐level mental health and substance use learning. YWHO's Minimum Data Set (MDS) contains more than 500 data elements that include repeated administration of standardized clinical measures to track clinical outcome changes over time, demographics, youth‐stated service and intervention goals, service experience and satisfaction and service provider reports of intervention modalities (i.e., SFBT, DBT, CBT, etc.) and youth's needs addressed. YWHO's data platform, MDS and data collection processes have been informed by the perspectives and preferences of YWHO's youth and family provincial advisory councils. Data used for evaluation and quality improvement are de‐identified. The YWHO‐PO and Network Leads have a data sharing agreement that sets out the privacy, legal and ethical terms and conditions governing the collection, use, modification, retention and disposal of data.

Table 1 provides the visit characteristics of youth included in the sample. Overall, 1851 youth provided data across 7348 visits, for an average of 3.97 visits per youth. During these visits, youth provided a total of 19,290 goals (an average 2.6 goals per visit or 10.4 total goals per youth). During this period, a total of 5819 youth visited YWHO hubs for services; however, not all hubs had fully implemented the GBO form, leading to variability in data collection.

**Table 1:** Visit characteristics of the study sample.

Variable	Youth *n* (%)
Total number of visits (visit frequency)	7348
Total youth	1851
Average visits per youth	3.97
Total number of goals	19,290
Mean goals per visit	2.6

### 4.3 Measures

#### 4.3.1 GBOs

GBOs are a measure of youth‐focused service needs [21]. At the start of a visit, regardless of treatment modality or intervention, youth are asked to identify one to three goals that reflect, in their own words, what they may want to achieve through engagement in YWHO services. Goals are identified by youth using an iPad, in response to the following prompt: *In coming to this service, what are some of the problems you want help with or goals you want to get to? (List up to three goals)*. Three open fields with corresponding rating scales are provided to youth at their first visit, and, at each subsequent visit more than 7 days later goals from the previous visit are pre‐populated into the goal fields, and youth can choose to either keep these goals or replace them with new ones.

#### 4.3.2 Goal Achievement

Complementing GBO data, a quantitative scale is provided for each goal for youth to rate how close they perceive they are to reaching each specific goal. Youth are asked: *How close are you to the goals you want to get to? On a scale from zero to ten, please indicate the number below that best describes how close you are to reaching your goal today*. An 11‐point scale (0–10) is provided along an axis, which is labelled ‘Goal Not Met’ on the left‐hand side above 0 and ‘Goal Reached’ on the right‐hand side above 10. Youth select the number they feel best reflects their goal achievement; however, completion of goals and their corresponding ratings are voluntary and some youth decide not to rate goals. To transform goal ratings into a single dependent variable in linear regression, a mean score across all goals was generated.

### 4.4 Analysis

#### 4.4.1 Content Analysis

A content analysis [31] was conducted on GBO data for the purpose of generating emergent codes [32] that could categorize data and provide descriptive findings using themes. Authors (D.C. and A.T.) independently coded the first third of the dataset (approximately 6500 goals) by organizing goals into ‘codes’ that categorize data [33]. After the first round of coding, authors (D.C. and A.T.) reviewed each other's analyses, highlighting any discrepancies or disagreements in both code development and assignment of codes to goals. Each of these discrepancies was discussed and resolved until a cohesive sub‐dataset was accomplished. This method is known as ‘constant comparison’ [31]. During the coding and reviewing process, it became apparent that several goals could be categorized across two codes (e.g. ‘I want to feel better about myself’ and ‘help get on track with eating again’). These goals were provided two codes to better reflect youth voices and capture the nuances in stated goals. However, this precluded any validity testing (such as inter‐rater reliability) as goals were no longer mutually exclusive across codes.

A total of 14 codes were generated from the preliminary analysis. The author A.T. then used this codebook to finish the analysis of the remaining data, which was checked again by the author D.C. Finally, codes for the entire dataset were collapsed into six main themes agreed upon by D.C. and A.T. The total number of goals for each theme was generated for distribution of data. Some goals were not written by youth in ways that were easily understood and/or lacked enough context to code reliably. These goals (*n* = 129) were categorized together and excluded from the analysis.

## 5 Results

### 5.1 Goal Themes

Content analysis of GBO data resulted in six themes. The theme *improve mental health* (including codes *improve mental health*, *reduce self‐harm and suicidal ideation* and *obtain diagnosis and assessment*) pertained to goals seeking support with mental health. Commonly, youth stated the need to integrate new techniques or strategies to help improve mental health, such as enhancing coping strategies and emotional regulation techniques and developing a better understanding of their thoughts, feelings and emotions. For example, one youth stated: ‘To gain coping skills that I can add on to my coping toolbox’ (Youth3100411). In addition, this theme included goals related to reducing self‐harm and suicidal ideation, such as ‘To talk so I don't feel like self‐harming’ (Youth10100064). Finally, goals pertaining to obtaining a professional diagnosis were included in this theme, such as ‘I want to try and get a diagnosis for anxiety and BPD’ (Youth8100396). The theme *improve mental health* accounted for over a third (42.7%) of all goals stated by youth.

The second theme, *connect to services*, reflected goals about support accessing a specific service related to medical, mental and/or physical health needs, as well as accessing community social supports, such as housing, employment, education and financial services. It included codes *access community social supports, service navigation* and *general help seeking*. For example, one youth stated: ‘Have a psychiatrist that understands my difficulties and can help me with my medications’ (Youth1010026), whereas another commented on education and employment‐related plans: ‘Help to graduate high school and explore either post‐secondary education or employment options afterwards’ (Youth2100819). Other youth articulated general help‐seeking goals such as needing to ‘talk to someone’ or learn about services offered at the hub. *Connect to services* was the second largest theme by goal at 20.9%.

*Intrapersonal development* included goals focusing on internal dynamics relating to personal growth and skill development, which covered two codes: *Skill development* and *personal growth*. *Skill development* pertained to goals focusing on developing tangible skills within specific domains, such as learning an instrument, doing art, and engaging in other creative endeavours, as one youth noted: ‘Learn to play at least four songs on the drums’ (Youth10100886). Similarly, youth commented on personal growth via improving self‐understanding and self‐worth by learning about their thought and behaviour patterns and integrating healthier habits. One youth wrote ‘Create and re‐enforce better and long lasting goals and habits within my daily life’ (Youth4100130). *Interpersonal development* reflected goals focusing on improving relationships by adopting and practicing new and healthier behaviours, or working through conflict and abuse within relationships. This included codes *manage interpersonal difficulties* and *improve communication*. For example, one youth stated their goal was ‘Not letting a new relationship be my only source of happiness. Being able to be around couples without hating myself and feeling jealous’ (Youth6100173). Other goals sought to improve communication through the development of skills and ability to articulate thoughts and feelings to others in an effective way, such as learning how to speak to parents calmly, or brainstorming how to express something important to a close family member or friend. One youth stated ‘To stop or reduce arguing with my partner in general’ (Youth10100060). *Intrapersonal development* (15.3%) and *interpersonal development* (12%) had similar distribution across youth goal data.

The theme *improve physical health* included youth seeking physical health resources and services, such as support with eating and nutrition, medication management, and addressing physical health symptomology. This encompassed codes *improve physical health* and *support for medication management*. Within this dataset, eating behaviours were often cited by youth; one youth stated, ‘Eating better to sustain myself’ (Youth2100903). Other youth cited engagement in sports or other physical activities as a reason for hub engagement. Medication management was also common; one youth indicated they sought to ‘find the right combination of medication for me’ (Youth6100470). Additionally, 5.4% of youth indicated a goal relating to *improve physical health*.

Finally, *address substance use and other addictions* included goals about seeking support with problematic substance use or reducing substance use by integrating new techniques or strategies, including codes *address substance use* and *address addiction behaviours*. One youth stated, ‘Figure out why I am doing so poorly and if it is related to chemical problems or situational problems or both. And if it is chemical problems than I want to figure out how I can fix things’ (Youth6100223). Other youth touched on addictive behaviours, such as ‘porn addiction’ and ‘gambling’. *Address substance use and addiction* was the least common theme across youth goals at 3%. See Table 2 for a summary of goal distribution across themes.

**Table 2:** Distribution of goals across categories.

Theme	No. of goals, *n* (%)
Improve mental health	9294 (42.7)
Connect to services	3733 (20.9)
Intrapersonal development	2838 (15.3)
Interpersonal development	2442 (12.0)
Improve physical health	1056 (5.4)
Address substance use/addiction	573 (3.0)

### 5.2 Descriptive Statistics

Table 3 provides descriptive statistics for visits and goal ratings. Overall, youth visited the hub an average of 10.8 times (i.e., ‘total visits’), with a standard deviation of 14.1. The mean goal achievement for the entire sample (i.e., ‘total goal rating’), including all themes, was 3.7. The goal theme with the highest achievement was *connect to services* (*M* = 4.4), followed by *improve mental health* (*M* = 3.9). *Interpersonal development* was the third highest (*M* = 3.5), and *address substance use/other addiction* and *interpersonal development* had a similar mean achievement rating of 3.1. *Improve physical health* was the lowest‐rated theme (*M* = 2.9). Standard deviations for goal achievement ranged from 2.5 to 1.7.

**Table 3:** Descriptive statistics.

Variable	Number *M* (SD)	Goal rating *M* (SD)
Total visits	10.8 (14.1)	
Total goal rating (i.e., goal achievement)		3.7 (2.3)
Connect to services		4.4 (2.1)
Improve mental health		3.9 (1.7)
Interpersonal development		3.5 (1.7)
Intrapersonal development		3.1 (1.9)
Address substance use/addiction		3.1 (2.1)
Improve physical health		2.9 (2.5)

## 6 Discussion

The analyses presented in this article were conducted using a large sample of goals from young people and based on data that are collected routinely from youth accessing IYS hubs. Specifically, we explored the type and frequency of goals set by young people receiving services within YWHO with the aim of understanding goal achievement as a function of service engagement. Typically, youth provided multiple goals per visit (*M* = 2.6), which is congruent with previous research from youth mental health services where young people report more than one reason for seeking services [24, 25]. Although youth articulated working on goals across a range of diverse areas, not surprisingly, mental health‐related goals were most common. Similar IYS services such as *headspace* in Australia and *Jigsaw* in Ireland report most young people seek help for concerns with issues related to feelings such as anxiety and worry, anger and thoughts about hurting oneself and physical health issues [24, 25, 34]. This highlights the importance of low‐barrier, youth‐friendly, integrated services that address multiple needs across a continuum of intensity within community‐based settings such as IYS [7, 12]. Research on the GBO in youth settings has found that young people believe goals help prioritize intervention targets (Bromley and Westwood, 2013) and that there is a meaningful improvement in goal progress after engaging in services [24]. Goal setting may be especially relevant for young people accessing youth mental health services as research has found that any form of goal setting within mental health service delivery reduces the risk of youth disengaging early from treatment [25] and helps young people to feel more included in their care [35].

Findings from the content analysis generally followed previous research seeking to develop taxonomic frameworks for goal achievement. For example, general themes mostly aligned with those of Rupani et al. [27], which included goals relating to emotional regulation, interpersonal development and personal growth. Similarly, when comparing with the BIT‐G framework, several commonalities were apparent in terms of an emphasis on mental health, interpersonal relationships and personal growth [26]. However, both studies are limited to capturing treatment goals in psychotherapy settings, whereas YWHO services are holistic and address a broader array of needs beyond mental health and counselling (e.g., primary care, substance use, education, employment and culture).

It is noteworthy that about 21% of goals were related to support with service navigation as evidenced by the *connect to service* theme. This is promising, as many youth with mental health challenges do not seek help [8, 13, 36] and often report little knowledge of available services [37] or poor accessibility of services [13, 37, 38]. IYS models such as YWHO bring together mental health, substance use, primary care and other social and vocational services by addressing the full range of both needs and strengths of youth holistically. Internationally, research has identified that the IYS approach is effective in increasing service access to all youth, in particular to youth who may not have otherwise accessed services [13, 39].

The current findings also demonstrate that *addressing substance use concerns/other addictions* was the least common goal set by youth (3% of total goals). This is not surprising given that youth have shorter substance use histories and therefore often express fewer negative consequences related to their substance use, which may reduce their perceived need for services [40, 41]. For some youth, the stigmatization surrounding youth substance use [37, 40, 42] may be a factor in reducing young people's ability to recognize problems that may arise due to their substance use and why they may not initially identify substance use as a goal for service. It is also possible that some youth may not be ready to address substance use challenges when they present for service but rather are prioritizing other concerns such as those identified in the current study related to mental health, relationships, service access, etc. Consideration should therefore be given to ensure that service providers use techniques such as MI to establish safe and supportive relationships with young people to enhance their readiness and engagement to access substance use services.

## 7 Limitations

Goal achievement ratings were self‐reported by youth, and whether service providers/family caregivers would have agreed or disagreed on the type or achievability of goals set was unable to be validated. It is also unclear whether, for some youth, goals were set collaboratively with providers or family/caregivers This lack of clarity around whether the goals were purely youth‐driven or shaped by others can make it difficult to assess the extent to which the goals represent the youth's personal values, needs and aspirations. It may also introduce variability in the interpretation and coding of goals, as goals set collaboratively could differ in terms of specificity, feasibility and relevance compared to those solely identified by youth themselves. It was also beyond the scope of this article to examine the extent to which goal ratings were associated with self‐reported clinical outcomes. Future research should examine whether an idiographic tool such as the GBO is more capable than standardized measures of capturing relevant clinical change for youth.

Some goals were not written by youth in ways that were easily understood and/or lacked enough context to code reliably. These goals (*n* = 129) were categorized together and excluded from the analysis. This exclusion may have introduced a potential bias, as these goals could represent perspectives or experiences that were not captured in the final dataset. Additionally, the lack of clarity or context in these goals may reflect challenges in communication, understanding or the framing of goals by the participants, which could be important to consider when interpreting the results. Also excluded from the analysis were youth who did not provide GBO data. Although the sample size for the current study is quite high, it is not possible to ascertain if excluded youth would have changed the findings significantly. For example, youth who do not improve or achieve goal attainment may self‐exclude from providing GBO data. However, this is partially controlled by the data collection method, where staff provided youth with previous goal data and supported ongoing data collection. Service providers using the GBO in practice could benefit from employing additional strategies to enhance clarity and context in goal setting with youth.

## 8 Conclusion

There has been a lack of literature exploring the types and frequencies of goals being set by youth accessing IYS or other mental health services and examining the achievement of those goals throughout service delivery. This article outlined a quantitative analytical approach to investigating the goals, using the GBO tool, of youth seeking services within IYS. This included an exploration of the type of goals youth set and how those goals were rated across categories. In line with the core value of youth‐centered care within the YWHO service model, the use of the GBO tool allows youth to actively participate in setting their own assessment and outcome criteria, indicate with which areas they require support and wish to improve, and can potentially personalize and facilitate shared decision‐making within IYS settings.

## Author Contributions

**Deb Chiodo:** conceptualization, investigation, writing – original draft, methodology, validation, writing – review and editing, formal analysis, project administration. **Aaron Turpin:** conceptualization, investigation, writing – original draft, writing – review and editing, validation, methodology, formal analysis, data curation. **Janis Wolfe:** conceptualization, writing – original draft, writing – review and editing, project administration, validation. **Karleigh Darnay:** conceptualization, writing – original draft, writing – review and editing, project administration, validation. **Jo Henderson:** supervision, project administration, funding acquisition, investigation, conceptualization, writing – original draft, writing – review and editing, validation.

## Conflicts of Interest

The authors declare no conflicts of interest.

## Data Availability

The authors have nothing to report.
